# Resolving cryptic species complexes of major tephritid pests

**DOI:** 10.3897/zookeys.540.9656

**Published:** 2015-11-26

**Authors:** Jorge Hendrichs, M. Teresa Vera, Marc De Meyer, Anthony R. Clarke

**Affiliations:** 1 Insect Pest Control Section, Joint FAO/IAEA Division of Nuclear Techniques in Food and Agriculture, Vienna, Austria; 2 Cátedra Terapéutica Vegetal, Facultad de Agronomía y Zootecnia (FAZ), Universidad Nacional de Tucumán (UNT), San Miguel de Tucumán; Argentina; 3 Consejo Nacional de Investigaciones Científicas y Técnicas (CONICET), Argentina; 4 Royal Museum for Central Africa, Invertebrates Unit, Leuvensesteenweg 13, B3080 Tervuren, Belgium; 5 School of Earth, Environmental and Biological Sciences, Queensland University of Technology (QUT), GPO Box 2434, Brisbane, QLD 4001, Australia

**Keywords:** *Anastrepha fraterculus*, *Bactrocera carambolae*, *Bactrocera dorsalis*, *Ceratitis anonae*, *Ceratitis fasciventris*, *Ceratitis rosa*, *Zeugodacus cucurbitae*, integrative taxonomy, Sterile Insect Technique, sibling species

## Abstract

An FAO/IAEA Co-ordinated Research Project (CRP) on “Resolution of Cryptic Species Complexes of Tephritid Pests to Overcome Constraints to SIT Application and International Trade” was conducted from 2010 to 2015. As captured in the CRP title, the objective was to undertake targeted research into the systematics and diagnostics of taxonomically challenging fruit fly groups of economic importance. The scientific output was the accurate alignment of biological species with taxonomic names; which led to the applied outcome of assisting FAO and IAEA Member States in overcoming technical constraints to the application of the Sterile Insect Technique (SIT) against pest fruit flies and the facilitation of international agricultural trade. Close to 50 researchers from over 20 countries participated in the CRP, using coordinated, multidisciplinary research to address, within an integrative taxonomic framework, cryptic species complexes of major tephritid pests. The following progress was made for the four complexes selected and studied:

FAO/IAEA Co-ordinated Research Project

Sterile Insect Technique

*Anastrepha fraterculus* complex – Eight morphotypes and their geographic and ecological distributions in Latin America were defined. The morphotypes can be considered as distinct biological species on the basis of differences in karyotype, sexual incompatibility, post-mating isolation, cuticular hydrocarbon, pheromone, and molecular analyses. Discriminative taxonomic tools using linear and geometric morphometrics of both adult and larval morphology were developed for this complex.

*Bactrocera dorsalis* complex – Based on genetic, cytogenetic, pheromonal, morphometric, and behavioural data, which showed no or only minor variation between the Asian/African pest fruit flies *Bactrocera dorsalis*, *Bactrocera papayae*, *Bactrocera philippinensis* and *Bactrocera invadens*, the latter three species were synonymized with *Bactrocera dorsalis*. Of the five target pest taxa studied, only *Bactrocera dorsalis* and *Bactrocera carambolae* remain as scientifically valid names. Molecular and pheromone markers are now available to distinguish *Bactrocera dorsalis* from *Bactrocera carambolae*.

*Ceratitis* FAR Complex (*Ceratitis fasciventris*, *Ceratitis anonae*, *Ceratitis rosa*) – Morphology, morphometry, genetic, genomic, pheromone, cuticular hydrocarbon, ecology, behaviour, and developmental physiology data provide evidence for the existence of five different entities within this fruit fly complex from the African region. These are currently recognised as *Ceratitis anonae*, *Ceratitis fasciventris* (F1 and F2), *Ceratitis rosa* and a new species related to *Ceratitis rosa* (R2). The biological limits within *Ceratitis fasciventris* (i.e. F1 and F2) are not fully resolved. Microsatellites markers and morphological identification tools for the adult males of the five different FAR entities were developed based on male leg structures.

*Zeugodacus cucurbitae* (formerly Bactrocera (Zeugodacus) cucurbitae) – Genetic variability was studied among melon fly populations throughout its geographic range in Africa and the Asia/Pacific region and found to be limited. Cross-mating studies indicated no incompatibility or sexual isolation. Host preference and genetic studies showed no evidence for the existence of host races. It was concluded that the melon fly does not represent a cryptic species complex, neither with regard to geographic distribution nor to host range. Nevertheless, the higher taxonomic classification under which this species had been placed, by the time the CRP was started, was found to be paraphyletic; as a result the subgenus *Zeugodacus* was elevated to genus level.

## Introduction

Tephritid fruit flies (Diptera: Tephritidae) are among the world’s worst pests of agriculture, being of major economic importance in nearly all tropical, subtropical and temperate countries (Cavalloro 1983, White and Elson-Harris 1994). By laying their eggs directly into fruit, where the maggots feed and develop, these pest species cause enormous devastation to both food production and international trade in spite of often intensive insecticide applications. They are among the primary causes of poverty, malnutrition and poor production and trade in fresh horticultural commodities in large areas of tropical developing countries, impeding the development of lucrative and labour-intensive fruit and vegetable-based agroindustries in rural areas (Waterhouse 1993, Allwood and Leblanc 1996).

The study of the biology and management of tephritids requires significant international attention to overcome transboundary hurdles and to assist the global community in developing and validating more environment-friendly fruit fly suppression systems to support viable fresh fruit and vegetable production and export industries. Such international attention has resulted in the successful development and validation of a Sterile Insect Technique (SIT) package for the Mediterranean fruit fly, *Ceratitis capitata* (Wiedemann, 1824) (Dyck et al. 2005). R&D support for this pest species is diminishing due to successful integration of the SIT into area-wide integrated pest management (AW-IPM) programmes to manage *Ceratitis capitata* populations (Enkerlin 2005). On the other hand there is increased demand from Africa, the Asia-Pacific and Latin America to address other major tephritid species or groups of economic importance. Some of these major pest fruit fly species occur within cryptic species complexes that include taxonomically described species that may actually be geographical variants of the same species. Conversely, some fruit fly populations grouped taxonomically within the same pest species display different biological and genetic traits, including reproductive isolation, which suggest that they are different species (Clarke and Schutze 2014). This uncertain taxonomic status has important practical implications on the effective development and use of the SIT against such pest complexes where the species under mass-rearing is not the same as the population occurring in the target area. Uncertainty of taxonomic status can also result in the incorrect establishment of trade barriers for agricultural commodities that are hosts of pest tephritids.

The resolution of some of the taxonomic uncertainties that surround major cryptic species complexes is therefore critical both for integrated SIT application and for subtropical and tropical countries to overcome non-tariff trade barriers, enabling them to export their fresh fruit and vegetable commodities to international markets. In particular, it is essential that the sterile males from such species complexes produced in regional fruit fly rearing facilities and destined for release in different countries or regions are behaviourally fully compatible with the target native fruit fly pest populations in the various recipient regions (Cayol et al. 2002). If the taxomomic status of species complexes remained unresolved, it would be difficult or impossible to achieve this desirable goal.

To address these issues, a major international collaboration was initiated in 2010 under the auspices of the Joint Food and Agriculture Organization / International Atomic Energy Agency (FAO/IAEA) Programme on Nuclear Techniques in Food and Agriculture. This paper summarises the goals, achievements and results of this coordinated research project that are compiled in this special issue of Zookeys (2015, Special Issue 540).

## Approach

**Figure 1.**
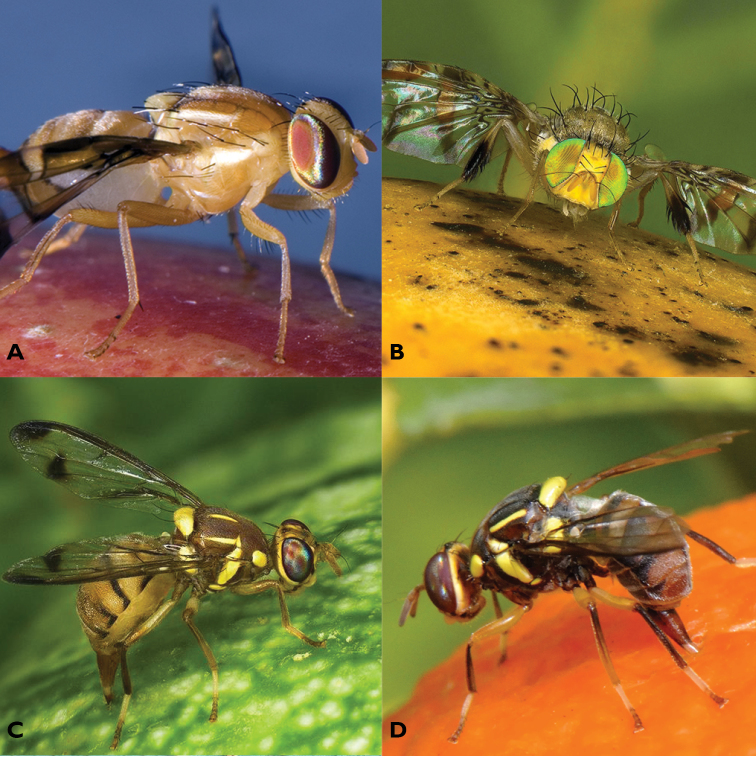
Habitus image of a representative of the four cryptic species complexes. **A** *Anastrepha fraterculus* **B** *Ceratitis rosa* (R2 type) **C** *Zeugodacus cucurbitae* **D** *Bactrocera dorsalis*. Photo credits: **A** Michal Hoskovec, **B** and **C** Antoine Franck, **D** Ana Rodriguez.

During a Consultants’ Meeting, held from the 6^th^ to 10^th^ of July 2009 in Vienna, Austria, the potential for conducting co-ordinated R&D in this area was assessed, and the major tephritid pest complexes were discussed and prioritised in terms of economic importance and potential for SIT application. Three complexes, the *Anastrepha fraterculus* complex (Latin America), the *Bactrocera dorsalis* complex (Asia and Pacific, Africa), and the *Ceratitis* FAR (= *Ceratitis anonae* Graham, 1908, *Ceratitis fasciventris* (Bezzi, 1920), *Ceratitis rosa* Karsch, 1887) complex (Africa) were confirmed to be of priority. The possibility that *Bactrocera cucurbitae* (Coquillett, 1899) (Asia and Pacific, Africa) also represents a species complex was evaluated and considered a lower, but still important, priority. In each of these groups (Figure 1), questions were raised concerning the validity of some of the described species, the capacity to diagnose described species, or the strong *a priori* evidence that unrecognised sibling taxa may occur.

A proposal for an FAO/IAEA Co-ordinated Research Project (CRP) on “Resolution of Cryptic Species Complexes of Tephritid Pests to Overcome Constraints to SIT Application and International Trade” was formulated and approved for the period 2010-2015. The specific objectives of this CRP were to define, using an integrative taxonomic approach (Schlick-Steiner et al. 2010), the species limits within the target complexes, and to develop robust species-specific diagnostic tools.

This international research network was operated under the IAEA Research Contract Programme and included 22 research teams. Other research teams also participated directly or indirectly and were fully funded by their institutions and governments. Overall close to 50 researchers from over 20 countries from all continents participated at one time or another during the six years of the CRP (2010-2015).

Research networks were established to (1) encourage close collaboration among institutes from developed and developing countries, (2) provide a forum for information exchange between scientists, and (3) embrace a focused approach to the development, capacity building and technology transfer of environment-friendly technologies. A worldwide network of partners provided representative samples of the fruit fly populations in order to assess the genetic diversity throughout the distributional ranges of the members of each complex. A generic protocol for collection and shipment of live and dead insects for vouchering, rearing, morphological and morphometric studies, chemical ecology and molecular assays was developed at the start of the CRP and used for distribution of material between the participating research units. Whenever possible, colonies of populations were established at the FAO/IAEA Agriculture and Biotechnology Laboratories in Seibersdorf, Austria, to be able to carry out field cage cross-mating studies that would not have been acceptable at other locations due to quarantine regulations and the risk of pest establishment.

During the implementation of the CRP, four Research Co-ordination Meetings (RCMs) were held to review research progress and to agree on future research directions and activities: the first RCM in Vienna, Austria from 2–6 August 2010, the second RCM in Brisbane, Australia from 31 January–3 February 2012, the third RCM in Tucumán, Argentina from 26–31 August 2013 and the fourth and final RCM in La Réunion, France from 1–5 June 2015.

## Situation Analysis at the Start of the CRP and Ouputs for Each Complex

### 1.1 *Anastrepha fraterculus* Complex Situation Analysis

The South American fruit fly, *Anastrepha fraterculus* (Wiedemann, 1830) s.l., is present in most countries of the Americas from the USA to Argentina (Hernández and Aluja 1993, Steck 1999, Zucchi 2007). Its centre of diversity is the South American subcontinent, where formerly it was thought to occurr in two, possibly unconnected bands: one along the western edge, including both highland and lowland areas of the Andean range, and the other along the east coast. However, recent data indicate its presence in parts of the Brazilian Amazon basin (Zucchi et al. 2011). It has been reported to infest about 110 host plants including major fruit crops (Norrbom and Kim 1988, Zucchi 2000, 2015, Norrbom 2004). The presence of this highly destructive pest results in quarantine restrictions for fruit export to many countries (Steck 1999).

The high levels of variability found among different populations throughout the geographical range of *Anastrepha fraterculus* led to the conclusion that it is a complex of cryptic species rather than a single biological entity (Stone 1942, Morgante et al. 1980, Malavasi and Morgante 1982, Solferini and Morgante 1987, Steck 1991, Steck and Sheppard 1993, Selivon 1996, Hernández-Ortiz et al. 2004). Differences have been reported based mainly on morphology, pest status and genetics (including karyotype, isozyme and molecular analyses); these are reviewed in Steck (1999) and some aspects discussed in subsequent studies (McPheron et al. 1999, Norrbom et al. 1999, Gomes Silva 2000, Smith-Caldas et al. 2001, Aluja et al. 2003, Hernández-Ortiz et al. 2004, Barr et al. 2005, Selivon et al. 2005, Goday et al. 2006, Selivon and Perondini 2007, Silva and Barr 2008, Prezotto 2008, Cáceres et al. 2009). However, in order to establish how much of this variation reflects population level variation, and how much reflects unrecognised cryptic species diversity, it is necessary to systematically correlate these genetic and morphological differences with the existence of reproductive isolation and other life history related traits (hosts, demography, etc).

Reproductive incompatibility has been reported both at pre- and post-zygotic levels between some *Anastrepha fraterculus* populations. At the pre-zygotic level, mating compatibility was evaluated among different populations from South America, involving lowland (Peru) and highland (Colombia) areas from the Andean region, and the south-eastern part of the continent (Brazil and Argentina). Most of the populations were shown to have some level of incompatibility with each other and thus appeared sexually isolated. Flies of different populations were often sexually active at different times of the day suggesting different sexual behaviour (Selivon 1996, Vera et al. 2006, Cáceres et al. 2009).

Post-zygotic studies between two populations from Brazil (Selivon et al. 1999) and between one Argentinean population and one Peruvian population (Cáceres et al. 2009) found partial hybrid inviability and sex ratio distortion confirming the existence of post-zygotic barriers. In the former case, cytological, isozyme and molecular studies revealed differences among groups (Malavasi and Morgante 1982, Selivon 1996, Selivon et al. 2005, Goday et al. 2006); while for the latter case, differences between groups were also found in terms of male sex pheromones and karyotypes (Cáceres et al. 2009).

The combined results of these studies suggested the existence of seven different biological entities referred to as: *Anastrepha* sp. 1 aff. *fraterculus* (Brazilian 1 morphotype) (Yamada and Selivon 2001), *Anastrepha* sp. 2 aff. *fraterculus* (Brazilian 2 morphotype) (Yamada and Selivon 2001), *Anastrepha* sp. 3 aff. *fraterculus* (Brazilian 3 morphotype) (Selivon et al. 2004), *Anastrepha* sp. 4 aff. *fraterculus* (Peruvian morphotype) (Selivon et al. 2004), *Anastrepha fraterculus* Mexican morphotype (Hernández-Ortiz et al. 2004), *Anastrepha fraterculus* Andean morphotype and *Anastrepha fraterculus* Venezuela coastal lowlands (Steck 1991).

Although previous studies provided strong evidence supporting the existence of several biological species, major knowledge gaps still existed in 2010. In particular, the described studies used different methodologies, did not use the same identified biological material and, most importantly, did not include all of the morphotypes. Therefore, in order to be able to formally describe and name these putative species, it was considered critical to apply a standardised, complete set of methodologies to all populations from the entire geographic distribution range in a comprehensive integrative taxonomy study. This would allow the characterization of each putative species and would provide sound diagnostic tools for addressing the related management and trade issues.

Definition of species limits and formal naming of these putative species will be relevant for plant protection authorities in determining which of them may or may not be quarantine pests. This would immediately allow some countries to gain access to international fresh fruit markets for those countries and commodities which can be determined to be outside the geographic and host range of correctly delimited *Anastrepha fraterculus* s.s. In addition, detailed studies on pest status, host range, economic impact and distribution would minimize any possible impact on trade between South American countries. Furthermore, knowing species boundaries and their levels of sexual compatibility within the complex would enable the implementation of the SIT.

### 1.2 Outputs on the *Anastrepha fraterculus* complex

Colonies from five *Anastrepha fraterculus* morphotypes (Mexican, Andean, Peruvian, Brazilian 1 and Brazilian 3) were established and used for behavioural, chemical, cytological, molecular and larval morphology studies. Linear and geometric morphometry were validated as tools for morphotype discrimination. Comprehensive morphometric studies supported the existence of eight morphotypes: the seven reported previously and a new Equadorian morphotype (Hernández-Ortiz et al. 2012, 2015).

Guidelines for performing mating compatibility field cage tests were developed. Reproductive compatibility studies were performed among the five morphotypes. Among the combinations studied, morphotypes were incompatible at the pre- and post-zygotic level (Rull et al. 2012, 2013, Devescovi et al. 2014). Male calling and courtship behaviour were recorded for four morphotypes. Sexual behaviour studies helped to identify behavioural characteristics that allowed distinct morphotype descriptions such as time of sexual activity, acoustical signals and sequence of courtship behaviour (Rull et al. 2012, 2013, Devescovi et al. 2014, Dias et al. in press). One post-copulatory study revealed differences in sperm storage and remating propensity between the Peruvian and Brazilian 1 morphotypes (Abraham et al. 2014). Hybrid females tended to mate with hybrid males and as a result Segura et al. (2011) suggested that hybridization is a possible speciation mechanism. In all combinations analysed, post-zygotic isolation was found to be weaker than pre-zygotic (Devescovi et al. 2014). *Wolbachia* was detected in several morphotypes (Cáceres et al. 2009, Lima 2015, Lima et al. unpublished data) and more in-depth studies aiming to characterize these strains are in progress.

The chemical profiles of the male pheromones and cuticular hydrocarbons of these five morphotypes were characterized as complex blends that were qualitatively and quantitatively unique for the different morphotypes (Břízová et al. 2013, Gonçalves et al. 2013, Vaníčková et al. 2012, 2015a,c,d, Milet-Pinheiro et al. 2015). The description of the mitotic karyotypes from the Mexican, Colombian and Equadorian morphotypes, from which information was absent or incomplete, allowed confirming that karyotypes are unique for each morphotype (Canal et al. unpublished data). The polytene chromosome map for one morphotype was constructed (Gariou-Papalexiou et al. unpublished data). Internal Transcriber Spacer 1 (ITS 1) was found to be a good molecular marker to identify different groups (Sutton et al. 2015). Microsatellites were developed (Lanzavecchia et al. 2014) and proved to be successful to discriminate among morphotypes (Lima et al. unpublished data, Manni et al. 2015). The phylogenetic relationships of Andean-Ecuadorian populations were determined with other molecular markers (Ludeña et al. 2011).

Based on all the collected evidence it is now possible to describe four morphotypes as new species with the exception of the three Brazilian morphotypes for which it is still necessary to solve problems with the unknown origin of the holotype male of *Anastrepha fraterculus* (Wiedemann) and the new Ecuadorian morphotype from which further studies are required for an integrative description (Hernández-Ortiz et al. 2015). A manuscript with the description of the new species is in preparation (V. Hernández-Ortiz personal communication) and improved diagnostic tools are now available based on morphology, molecular markers, chemical profiles, cytology and sexual behaviour.

The knowledge and gaps identified at the start of the CRP, as well as the progress made by the end of the CRP in addressing the gaps identified for the *Anastrepha fraterculus* complex are summarized in Table 1.

**Table 1.** Baseline and progress on the *Anastrepha fraterculus* complex.

Method	Knowledge at CRP start and gaps identified	Progress in addressing gaps identified	Output References
DNA Analysis	Only three gene regions studied for representatives of *Anastrepha fraterculus* s.l. populations (COI, 16S, and period genes). Other molecular markers have not been applied. There are no data for several of the morphotypes and the total number of specimens analysed so far is small. Microsatellite data are not yet available.	Extensive sampling from more than 70 populations from ten Latin American countries. COI data showed Colombian populations to be related to each other; Brazilian populations clustered in three clades, one of which included the population from Argentina. Mexico formed a separate clade. Ribosomal ITS 1 studies performed on populations from Andean, Brazilian and Mesoamerican regions. For the Andean region a total of 6 ITS 1 sequence variants in 4 groups were identified. Several microsatellite loci isolated and validated as markers in populations pertaining to at least three different morphotypes.	Ludeña et al. 2011, Lanzavecchia et al. 2014, Manni et al. 2015 this issue, Sutton et al. 2015 this issue, Lima 2015, Canal et al. (unpubl.), Lima et al. (unpubl.)
Cytology	Karyotypes described for Brazilian 1, 2, 3, Mexican and Peruvian morphotypes. No karyotype available for Andean, Ecuadorian and Venezuelan morphotypes. No polytene chromosome banding patterns described.	Karyotypes were described for the Andean and Ecuadorian morphotypes and completed for the Mexican karyotype. New detailed photographic polytene chromosome maps of Brazil 1 morphotype constructed; these maps can be the reference for comparative polytene chromosome analysis among different morphotypes).	Canal et al. (unpubl.); Giardini, Gariou-Papalexiou et al. (unpubl.)
Morphology	At least three morphotypes recognized (Andean, Brazilian 1 and Mexican) based on discriminant function analysis of aculeus, wing and mesonotum. Indications of four additional morphotypes. Egg morphology only described for three Brazilian morphotypes. None of the larval stages have been thoroughly described and compared among morphotypes.	Morphometric analysis involving 8 populations from Ecuador, 11 from Colombia and 23 from Brazil. Description of seven adult morphotypes using a multivariate approach published. A new 8^th^ morphotype was recognized from Ecuador. Linear and geometric morphometric analysis of mouthparts of third instar larvae from five morphotypes (Andean, Brazilian 1, Ecuadorian, Mexican and Peruvian) allowed discrimination among morphotypes. SEM observations on third instar larvae of Brazilian 1, Mexican, and Peruvian morphotypes. Egg morphology of several *Anastrepha* species from the *fraterculus* group described.	Dutra et al. 2011, 2012, Hernández-Ortiz et al. 2012, this issue, Perre et al. 2014, Canal et al. 2015 this issue
Sexual Behaviour	Male courtship and female responses within and among morphotypes only partially known. Mating behaviour described for only 3 morphotypes, with very different mating times during day in field cage trials and segregated leks. Pre-zygotic isolation detected among some populations, but mating compatibility not evaluated among all morphotypes. Pre-zygotic isolation factors largely unknown.	High levels of sexual isolation among Andean, Brazilian 1, Mexican and Peruvian morphotypes; the Andean mated at dusk, the Brazilian 1 and Mexican early in the morning, and the Peruvian around midday. Populations from southern Brazil and Argentina compatible, while southern, southeastern and northeastern Brazilian populations partially or completely incompatible among each other. Detailed behavioural analysis including calling and sound analysis showed corresponding differences between these populations. Hybrids presented an intermediate calling behaviour. Remating propensity and duration of the refractory period independent of male origin. Tendency for Peruvian females mated with Brazil 1 males not to allow sperm transfer.	Dias 2012, Rull et al. 2012, 2013, Abraham et al. 2014, Cladera et al. 2014, Devescovi et al. 2014, Dias et al. in press, Juárez et al. 2015 this issue, Vaníčková et al. 2015d this issue
Post-zygotic compatibility	Crosses between populations from Peru and Argentina and Brazil 1 and Brazil 2 resulted in reduced egg hatch, larval viability and distorted sex ratio. *Wolbachia* presence was confirmed for some populations, but its role in post-zygotic incompatibility not determined.	Compatibility among one population from Argentina and two from Brazil demonstrated, as well as among six Colombian ones. Cross-mating among Brazilian 1, Brazilian 3, Colombian, Mexican and Peruvian morphotypes resulted in no or unviable eggs, or with significantly lower hatch rate and sex ratio distortion than those laid by females mated with homotypic males. The crosses of some F_1_ x F_1_ males and females resulted in high fertility levels. *Wolbachia* studies expanded to additional populations.	Rull et al. 2012, 2013, Devescovi et al. 2014, Lima et al. (unpubl.)
Pheromone Components	Chromatograms of male borne volatiles for two morphotypes (Brazilian 1, Peruvian) and their hybrids available. The function of identified chemicals either alone or combined in eliciting attraction of conspecific females unknown.	The male borne volatiles from Andean, Brazilian 1 and Brazilian 3 morphotypes were characterized. Gas chromatography – electroantenography (GC-EAD) of females from Andean, Brazilian 1, Brazilian 3 and Peruvian morphotypes showed some similarities but also specificities among morphotypes in the antennal active compounds.	Břízová 2011, Vaníčková 2012, Břízová et al. 2013, Zykova 2013, Vaničkova et al. 2015a this issue, Kalinova (unpubl.)
Cuticular hydrocarbons	Cuticular hydrocarbon composition known only from one Argentine and one southern Brazil population.	Cuticular hydrocarbons from males and females from various populations of Andean, Brazilian 1, Brazilian 3, Mexican and Peruvian morphotypes were characterised, showing significant differences.	Vaníčková 2012, Vaničkova et al. 2012, Vaničkova et al. 2015a,c this issue
Distribution	*Anastrepha fraterculus* s.l. is widely distributed from southern Texas to northern Argentina, but the detailed distributions of morphotypes largely unknown. Also elevational transects in Andean countries lacking and needed to determine limits of highland and lowland morphotypes.	Mexican morphotype extends from Mexico to Central America; Venezuelan morphotype occurs only in the Caribbean lowlands of Venezuela; Andean morphotype in the Venezuelan and Colombian highlands (above 900 m elevation); the Peruvian morphotype in coastal areas of Ecuador and Peru, the Ecuadorian morphotype in the inter Andean Valleys in Ecuador and the southeastern Andean Valleys in Peru; Brazilian 3 morphotype in the northeastern coastal and southeastern regions from Brazil; Brazilian 1 morphotype in Argentina and southern Brazil.	Zucchi et al. 2011, Hernández-Ortiz et al. 2012, Perre et al. 2014, Hernández-Ortiz et al. 2015 this issue
Host Range	Host lists for *Anastrepha fraterculus* s.l. have been published for several countries, but these have not been associated with the various morphotypes. Host ranges for most morphotypes are largely unknown.	In areas with only one morphotype (Argentina, Mexico and Central America, highland Colombia-Venezuela), host ranges are being updated based on existing records. A host list was established for Colombia and new host plant information obtained in Bolivia, Ecuador and Peru. The host list for Brazilian morphotypes was updated to 110 hosts.	Castañeda et al. 2010, Zucchi 2015

### 2.1 *Bactrocera dorsalis* Complex Situation Analysis

Across Asia and the Pacific the fruit fly subfamily Dacinae contains some 47 recognised pest species (Drew and Romig 2013). Of these, eight were recognized within the *Bactrocera dorsalis* complex, with some being the most economically damaging of all pest species within the subfamily (Drew and Hancock 1994, Clarke et al. 2005). Losses caused by *Bactrocera dorsalis* complex species include destruction of crops, restriction of international trade, and the establishment of a range of quarantine and regulatory activities carried out by various regional governments.

Background research on these flies has generated data on diagnostics, field surveillance, quarantine strategies, field pest control, and market access protocols (e.g. Tan and Nishida 1996, 1998, Muraji and Nakahara 2002, Naeole and Haymer 2003, Smith et al. 2003, Armstrong and Ball 2005). But the key knowledge gap of the *Bactrocera dorsalis* complex was a lack of consensus on species limits of the major pest species in the complex, particularly *Bactrocera dorsalis* s.s. (Hendel, 1912), *Bactrocera papayae* (Drew & Hancock, 1994), *Bactrocera philippinensis* (Drew & Hancock, 1994), *Bactrocera carambolae* (Drew & Hancock, 1994) and *Bactrocera invadens* (Drew, Tsuruta & White, 2005) (Clarke et al. 2005, Drew et al. 2005, Wee and Tan 2005, Ebina and Ohto 2006, Drew et al. 2008). Failure to resolve the taxonomic status of the members of this complex prevented further development towards SIT integration into AW-IPM programmes against these pest insects and limited international horticultural trade.

Background research on the taxonomy of the *Bactrocera dorsalis* complex has been unable to provide definitive identification of some species (Clarke et al. 2005). This has confounded collecting associated host plant records and defining geographic distributions. It was considered absolutely essential that the species be accurately identified to be able to apply AW-IPM field programmes that include a SIT component. Because the trade implications and response systems to detections and/or incursions are different for all members in the complex, “near-enough” identification is, unfortunately, not good-enough. Consequently countries have difficulty overcoming the phytosanitary barriers to export-trade to major importers such as Australia, Japan, Europe, New Zealand, South Africa, and the USA. Another severe problem would arise if one member of the complex is detected or becomes established in a country, but is unable to be differentiated from others in the complex. In this case that country would then be forced to admit that all members of the complex may in fact be present, which would result in extended trade embargoes.

Therefore a comprehensive integrative taxonomy approach involving biological, morphological, chemo-ecological and molecular studies of the various members of the *Bactrocera dorsalis* complex were needed to: (1) resolve species limits by seeking a consensus result from different tests; (2) examine congruence between data from the different approaches to either support taxonomic revision or retain existing species status; and (3) develop robust diagnostic tools for the identified species.

### 2.2 Outputs on Five Priority Species in the *Bactrocera dorsalis* Complex

Quantified cross-species field-cage mating trials, as described in the FAO/IAEA/USDA Quality Control Manual (FAO/IAEA/USDA 2014), were completed for *Bactrocera dorsalis* s.l. populations from China, Kenya, Malaysia, Pakistan, the Philippines, Suriname and Thailand. Results presented in Schutze et al. (2013), Bo et al. (2014), and Chinvinijkul et al. (2015) demonstrated pre- and post-zygotic mating compatibility between all target taxa except for crosses involving *Bactrocera carambolae*, which always showed some level of sexual isolation from the other taxa. Post-zygotic isolation tests up to three generations were carried out for crosses involving *Bactrocera dorsalis* s.s. (China and Pakistan) and *Bactrocera invadens*; no evidence for hybrid incompatibility was detected (Bo et al. 2014).

Chemical components and ratios of sex pheromone stored in male rectal gland and emitted during courtship after feeding on methyl eugenol (ME) were determined qualitatively and quantitatively for *Bactrocera dorsalis* s.s., *Bactrocera invadens*, *Bactrocera papayae* and *Bactrocera philippinensis*. The four ccomplex members had identical volatile emission profiles and rectal pheromonal components consisting of 2-allyl-3,4, dimethoxyphenol (DMP) and E-coniferyl alcohol (E-CF) (Tan et al. 2011, 2013), and the ratios of DMP: *E*-CF were not significantly different between the different members. Probit analysis showed that the responsiveness of these four members to ME was similar as their ED_50_ values (= dose at which 50 % of the population responded) were not significantly different (Hee et al. 2015a). However, differences were found for *Bactrocera carambolae*.

Wing shape variation analysed through geometric morphometrics was used for the first time in the *Bactrocera dorsalis* complex (Schutze et al. 2012a,b, Krosch et al. 2013). Variation in wing shape proved to be extremely informative in interpreting variation within the *Bactrocera dorsalis* complex.

Genetic, cytogenetic and molecular analyses of *Bactrocera dorsalis* complex specimens collected across the geographical range were carried out in participating laboratories in Asia, Australia and Europe:

*Cytogenetics*: One of the objectives was to identify and evaluate cytogenetic tools that could help to resolve the taxonomic status of the five taxa under study, focusing on chromosomal rearrangements, especially inversions. For this purpose, mitotic and polytene chromosomes were analysed from colonized specimen representing *Bactrocera dorsalis* s.s., *Bactrocera papayae*, *Bactrocera philippinensis*, *Bactrocera invadens* and *Bactrocera carambolae*. Analysis of mitotic karyotypes could not detect any differences among these five taxa (Yesmin and Clyde 2012, Yesmin 2013, Augustinos et al. 2014, Augustinos et al. 2015), showing that all had the typical *Bactrocera dorsalis* s.s. karyotype as previously described by Hunwattanakul and Baimai (1994). Polytene chromosome maps were developed for the first time of a member of the *Bactrocera dorsalis* complex, i.e. *Bactrocera dorsalis* s.s. (Zacharopoulou et al. 2011a). Subsequent analysis showed that the five members of the complex do not present any chromosomal rearrangements that could be used as diagnostic characters and therefore these taxa can be regarded as homosequential (Augustinos et al. 2014, 2015). Although *Bactrocera carambolae* presented the same mitotic and polytene chromosome karyotype as the other members of the complex, the presence of a high number of minor asynapses in F_1_ hybrids of *Bactrocera dorsalis* s.s. × *Bactrocera carambolae* crosses may indicate the presence of small differences in the chromosomal organization among the parental entities. However, these observations cannot be regarded as diagnostic at the species level (Augustinos et al. 2014).

*Microsatellites*: Microsatellite DNA markers derived from *Bactrocera dorsalis* s.s. were tested on populations of *Bactrocera dorsalis* s.s. from Bangladesh, Cambodia, China (multiple populations), Hawaii (two populations), Laos, Malaysia, Taiwan, and Thailand (multiple populations) (Shi et al. 2012, Krosch et al. 2013, Aketarawong et al. 2011, 2014a). The same set of markers combined with microsatellite markers derived from *Bactrocera papayae* were used to compare populations of *Bactrocera dorsalis* and *Bactrocera papayae* from the Thai/Malay Peninsula (Krosch et al. 2013, Aketarawong et al. 2014b). No genetic isolation was found between the *Bactrocera dorsalis* and *Bactrocera papaya* populations, supporting the hypothesis that both are the same entity. On the other hand, microsatellite markers, which amplify for *Bactrocera carambolae* and *Bactrocera dorsalis*, showed different genetic clusters between these two species, although admixture populations were observed. Admixture is evidence that some gene flow (i.e. hybridisation) may occur in the field between these species (Aketarawong et al. 2015).

*Haplotype analysis*: CO1 haplotype networks showed that common haplotypes were shared between *Bactrocera dorsalis*, *Bactrocera papayae*, *Bactrocera philippinensis* and *Bactrocera invadens*, but not with *Bactrocera carambolae* (Schutze et al. 2012b, 2015a). This supports the hypothesis that the first four taxa are a single biological species, while *Bactrocera carambolae* is distinct.

*Phylogenetic analysis*: A phylogenetic study using six neutral genetic markers found that *Bactrocera carambolae* could be resolved as a monophyletic clade from the other four target species, which were mixed together as an unresolved comb (Boykin et al. 2014, Schutze et al. 2015a).

Based on genetic, cytogenetic, pheromonal, morphometric and behavioural data, which repeatedly showed no or only minor variation between *Bactrocera dorsalis*, *Bactrocera invadens*, *Bactrocera papayae*, and *Bactrocera philippinensis*, formal taxonomic name changes were made. *Bactrocera philippinensis* was made a junior synonym of *Bactrocera papayae* by Drew and Romig (2013). Subsequently, also *Bactrocera papayae* and *Bactrocera invadens* were synonymised with *Bactrocera dorsalis* (Schutze et al. 2015b, Hee et al. 2015b), while the status of *Bactrocera carambolae* has not been altered. This means that only *Bactrocera dorsalis* and *Bactrocera carambolae* remain as scientifically valid names. The name changes have been widely accepted by national and regional plant protection organizations around the world, the Secretariat of the International Plant Protection Convention and the FAO (http://www.fao.org/news/story/en/item/262972/icode/).

In the works of Drew and Romig (2013), San Jose et al. (2013) and Schutze et al. (2015b) new morphological descriptions of the target taxa are provided. However, the use of morphology alone is not sufficient for definitive diagnosis of *Bactrocera carambolae* and *Bactrocera dorsalis*, but molecular and pheromone markers are now available to distinguish them. Molecular protocols using neutral genetic markers to distinguish the two species from each other, and from other closely related taxa, are provided in Boykin et al. (2014). Microsatellite markers which amplify for both species and which are used in population genetic studies, are provided in Aketarawong et al. (2015). The Y-specific marker will also separate the two species. For adult flies, the presence of the ME metabolites DMP and *E*-CF in the male rectal gland following ME feeding can be used to discriminate *Bactrocera dorsalis* from *Bactrocera carambolae* (which produces only *E*-CF) (Tan and Nishida 1996, Wee et al. 2007, Wee and Tan 2007).

The knowledge and gaps identified at the start of the CRP, as well as the progress by the end of the CRP in addressing the gaps identified for the *Bactrocera dorsalis* complex are summarized in Table 2.

**Table 2.** Baseline and progress on the *Bactrocera dorsalis* complex.

Method	Knowledge at CRP start and gaps identified	Progress in addressing gaps identified	Output References
DNA Analysis	There is no adequate and consistent sample coverage for the five target species. Nuclear ribosomal ITS1+2 diagnostic for separating *Bactrocera carambolae* from remaining four species. Mitochondrial DNA markers show no clear distinction between currently defined species. Microsatellite sequences available as well as nuclear coding gene data for 16 loci. Lack of discriminatory characters means that either they are yet to be discovered, or such characters do not exist and the species are the same.	Significantly improved sample coverage, including from China, Indian subcontinent, Myanmar, Indo/Malay Archipelago, and African populations. Additional diagnostic genetic markers (mitochondrial, ribosomal and nuclear genes) developed, but they do not discriminate between four of the five species; *Bactrocera carambolae* forms a monophyletic group according to COI and ITS1. A full multigene phylogenetic analysis published. Microsatellites indicate origin of *Bactrocera dorsalis* is China. Y specific primers have been developed and differences of the amplified sequences have been found between *Bactrocera dorsalis* and *Bactrocera carambolae*, but not for *Bactrocera dorsalis*, *Bactrocera papayae*, *Bactrocera philippinensis* and *Bactrocera invadens*. Combined genetic results cannot consistently separate *Bactrocera dorsalis*, *Bactrocera papayae*, *Bactrocera philippinensis* and *Bactrocera invadens*. Unique markers have been identified for *Bactrocera carambolae*.	Aketarawong et al. 2014a, 2014b, 2015 this issue, Boykin et al. 2014, Leblanc et al. 2015 this issue
Cytology	Studies on mitotic karyotypes identified several forms within the *Bactrocera dorsalis* complex, but did not definitively distinguish between putative species. Further polytene maps are needed to allow distinguishing between putative species. Polytene mapping for these species could be linked with genomic data.	Cytological evidence, neither on mitotic nor on polytene chromosomes can discriminate between the five target taxa, therefore these taxa can be regarded as homosequential. Hybrid analysis also shows no chromosomal structural differences; only minor asynapses were observed in a few hybrids between *Bactrocera dorsalis* and *Bactrocera carambolae*. *In situ* hybridization of the six unique sequences shows no evidence for the presence of chromosomal rearrangements. However, analysis of mitotic and polytene chromosomes from both *Bactrocera kandiensis* and *Bactrocera tryoni* clearly differentiates these taxa from *Bactrocera dorsalis* s.s.	Zacharopoulou et al. 2011a, Zacharopoulou and Franz 2013, Augustinos et al. 2015 this issue
Genomics	Unpublished Hawaiian *Bactrocera dorsalis* s.s. genome. Transcriptomics under way for *Bactrocera dorsalis* s.s., *Bactrocera philippinensis* and *Bactrocera carambolae*, potential of some markers for these species.	Public web portal for accessing the current scaffold and contig structure of the Hawaiian *Bactrocera dorsalis* s.s. genome. The genome for *Bactrocera tryoni* and draft genomes for *Bactrocera neohumeralis* and *Bactrocera jarvisi* have been published as reference genomes. Comparative transcriptome data for *Bactrocera dorsalis* s.s., *Bactrocera papayae*, *Bactrocera philippinensis*, *Bactrocera carambolae* and *Bactrocera invadens* have been analysed for ‘species’ specific SNPs. No specific SNPs could be identified.	Gilchrist et al. 2014, Armstrong et al. (unpubl.)
Morphology	No consensus on species limits of the major *Bactrocera dorsalis* complex pest species. Explicit intra-specific population-level variation in both external and internal morphological characters. Therefore for some species unable to provide definitive identification of specimens. Egg and immature morphology not investigated for *Bactrocera dorsalis* complex species.	*Bactrocera invadens vs Bactrocera dorsalis* s.s. colour morphs described and the range of morphological variation assessed from across their geographic ranges. Crosses between colour morphs (pale brown and black scutum) of *Bactrocera dorsalis* from Pakistan confirm that scutum colour morph is a simple qualitative genetic trait. Morphological pattern variation in *Bactrocera dorsalis* s.s has been assessed for flies fed varying quantities of food (standard lab diet). No clear correlation was observed. Egg and immature material has been gathered and is being included as part of a major project describing the immature stages of tephritids.	Leblanc et al. 2013, Leblanc et al. 2015 this issue, Schutze et al. 2015a
Morphometrics	Large number of morphometric studies for *Bactrocera dorsalis* complex species; often impossible to separate between and within population variation in morphometric traits. Insufficient understanding of relative environmental/genetic influences on morphometric phenotype. Morphometric approaches have rarely been linked adequately with other morphological or genetic approaches.	Geometric morphometric wing shape data are consistent with *Bactrocera dorsalis* s.s., *Bactrocera invadens*, *Bactrocera papayae* and *Bactrocera philippinensis* representing the same species which displays strong isolation-by-distance patterns within SE Asia. All ‘outgroup’ species resolve strongly from the *Bactrocera dorsalis* complex species. Aedeagi from a latitudinal gradient from northern Thailand to Peninsular Malaysia for *Bactrocera dorsalis* s.s. and *Bactrocera papayae* show a significant and continuous latitudinal cline from north to south, with northern Thailand flies the shortest and Malaysian flies possessing the longest aedeagi. Morphometrics of genitalia, head width and wings have been undertaken for *Bactrocera dorsalis* s.s. reared under different larval densities.	Schutze et al. 2012a, Schutze et al. 2012b, Krosch et al. 2013, Schutze et al. 2015a
Sexual Behaviour	Knowledge of some mating compatibility for individual species crosses from isolated studies. Lack of comparative mating compatibility studies across populations/species from across their geographic range acquired under equivalent semi-natural conditions.	Comparative field cage studies demonstrating complete pre- and post-zygotic compatibility among *Bactrocera dorsalis* s.s., *Bactrocera invadens*, *Bactrocera papayae* and *Bactrocera philippinensis*; hybrid offspring obtained between crosses are also viable. *Bactrocera carambolae* shows partial pre-and post-zygotic incompatibility with *Bactrocera dorsalis* s.l.. Following methyl-eugenol (ME) feeding by males *Bactrocera dorsalis* and *Bactrocera carambolae*, relatively sexual incompatibility remained in cross-mating field cage studies. No compatibility under same conditions with outgroups.	Chinvinijkul et al. 2015 this issue; Schutze et al. 2015c, this issue
Sensitivity to methyl-eugenol (ME)	Male responsiveness to ME varied between *Bactrocera dorsalis*, *Bactrocera papayae* and *Bactrocera carambolae*. Initial feeding on high-concentration ME reduced response to subsequent exposure in *Bactrocera dorsalis* s.s. The male mating advantage seen following ME-feeding begins later for *Bactrocera carambolae* relative to *Bactrocera dorsalis*. Lack of specific knowledge of the ME response of *Bactrocera philippinensis* and *Bactrocera invadens*. Species sensitivity to ME across the four species has not been compared.	Probit analysis on the males’ sensitivity to ME showed no significant differences in the ED_50_ between *Bactrocera dorsalis*, *Bactrocera papayae*, *Bactrocera philippinensis* and *Bactrocera invadens*. Electrophoretic analyses of the male antennal protein extracts for *Bactrocera dorsalis*, *Bactrocera papayae*, *Bactrocera philippinensis* and *Bactrocera invadens* also showed no differences in the protein electropherogrammes. This also included that of males exposed to ME.	Hee et al. 2015a this issue
Pheromone Components	Phermononal components following ME consumption identical in *Bactrocera dorsalis* s.s. and *Bactrocera papayae*. Endogenous pheromone components different between *Bactrocera carambolae* and both *Bactrocera dorsalis* and *Bactrocera papayae*. Pheromone components of *Bactrocera philippinensis* and *Bactrocera invadens* so far not been characterised. ME metabolites (i.e. DMP and *E*-CF) presence in male rectal gland derived from ME feeding can be used to discriminate against *Bactrocera carambolae* (which produces only *E*-CF).	Pheromone components post-ME feeding for *Bactrocera invadens* and *Bactrocera philippinensis* males identical to those recorded for *Bactrocera dorsalis* and *Bactrocera papayae*, but different to those of *Bactrocera carambolae*. Pheromone components in male rectal glands and volatile emissions virtually identical among *Bactrocera dorsalis*, *Bactrocera papayae*, *Bactrocera philippinensis* and *Bactrocera invadens*, but distinctive as compared to *Bactrocera carambolae*. Ratios of pheromonal components (DMP: *E*-CF) quantified from male rectal gland (in storage) and volatile emission following ME consumption were not significantly different between *Bactrocera dorsalis*, *Bactrocera papayae*, *Bactrocera philippinensis* and *Bactrocera invadens*. Endogenously produced pheromone constituents confirmed as unique marker for *Bactrocera carambolae* (i.e. 6-oxo-1-nonanol) to enable species separation from *Bactrocera dorsalis* s.l.	Hee et al. 2015a this issue, Tan et al. 2011, Tan et al. 2013
Cuticular hydrocarbons	No cuticular hydrocarbons of the *Bactrocera dorsalis* complex so far studied.	The identification and quantification of cuticular hydrocarbons of males and females of *Bactrocera philippinensis*, *Bactrocera papayae*, *Bactrocera dorsalis*, *Bactrocera invadens* and *Bactrocera carambolae* was performed on the GC×GC/TOFMS. Quantitative as well as qualitative CHC profile differences were found between sexes. Female profiles show high amount of short-chained hydrocarbons, not male profiles.	Kalinova et al. (unpubl.)
Distribution	Collection locality considered ‘species character-state’ in many operational keys. For example, *Bactrocera dorsalis* within Thailand is restricted to central/northern Thailand and *Bactrocera papayae* to southern Thailand, but zone of transition between *Bactrocera dorsalis* s.s. and *Bactrocera papayae* on the Thai/Malay Peninsula not confirmed. Endemic range of *Bactrocera invadens* is not known.	Two independent studies show there is no zone of transition between *Bactrocera dorsalis* s.s. and the former *Bactrocera papayae* on the Thai/Malay Peninsula based on population genetic data (e.g. Fst values and demonstrated gene flow) and morphological data, supporting a continuum rather than different species. Morphological and wing shape analysis suggests that the native range of invasive African *Bactrocera dorsalis* (formerly *Bactrocera invadens*) need not have been restricted to Sri Lanka but may have been more widely distributed across the Indian subcontinent. *Bactrocera dorsalis* is now recognised as a naturally wide-spread and highly invasive species which occurs across sub-Saharan Africa, across the Indian sub-continent to Asia and into the South Pacific.	Krosch et al. 2013, Aketarawong et al. 2014b, Schutze et al. 2015a
Host Ranges	Well documented for most pest populations being tested, but not yet fully for *Bactrocera philippinensis* and *Bactrocera invadens*.	A single host list is being consolidated, which covers *Bactrocera dorsalis* and the former *Bactrocera papayae*, *Bactrocera invadens* and *Bactrocera philippinensis*.	Luc Leblanc et al. (unpubl.)

### 3.1 *Ceratitis* FAR Complex Situation Analysis

The Afro-tropical fruit flies *Ceratitis fasciventris*, *Ceratitis anonae* and *Ceratitis rosa* (i.e. the *Ceratitis* FAR complex), together with *Ceratitis capitata* and *Ceratitis cosyra*, are considered major horticultural pests of that region (White and Elson-Harris 1994, De Meyer 200la). These species are of quarantine significance (EPPO/CABI 1997) as they are highly polyphagous and damage a wide range of unrelated wild and cultivated crops (De Meyer et al. 2002), resulting in enormous economic losses wherever they occur (Barnes 2000, De Meyer 2001b). They have different distribution patterns that partially overlap, resulting in sympatric occurrence in particular areas.

*Ceratitis rosa*, *Ceratitis fasciventris* and *Ceratitis anonae* were considered the three members of the *Ceratitis* FAR species complex (Virgilio et al. 2007a, 2007b). Taxonomically, *Ceratitis fasciventris* was initially considered a variety of *Ceratitis rosa* (Bezzi 1920) but has recently been recognized as a different entity with species status (De Meyer 2001a).

Unlike *Ceratitis capitata*, which has over the last century spread from its home range in East Africa and attained an almost world-wide distribution (Fletcher 1989, White and Elson-Harris 1994, http://www-naweb.iaea.org/nafa/news/images/Distribution-Mediterranean-fruit-fly-Ceratitis-capitata.jpg), *Ceratitis rosa*, *Ceratitis fasciventris* and *Ceratitis anonae* have so far not been reported outside the African mainland (except for La Réunion and Mauritius) but are potentially invasive.

Due to the difficulty in distinguishing morphologically some members of the complex, especially females, a number of molecular approaches for species recognition were used (Baliraine et al. 2004, Barr et al. 2006, Virgilio et al. 2008). However, these diagnostic tools remained inadequate for quarantine purposes and much more robust molecular markers were needed.

### 3.2 Outputs for *Ceratitis* FAR Complex

Development of molecular diagnostics, using microsatellites (Delatte et al. 2013), revealed a more complex structure than the mere existence of three entities within the *Ceratitis* FAR complex. Five genotypic groups were identified (Virgilio et al. 2013) and later confirmed by morphological differences of the males (De Meyer et al. 2015a). Morphological diagnostics for male specimens of the five entities, called R1, R2 (*Ceratitis rosa* type 1 and 2), F1, F2 (*Ceratitis fasciventris* type 1 and 2) and A (*Ceratitis anonae*) were developed. Morphometric diagnostics using wing landmarks were developed for both sexes to a certain extent (Van Cann et al. 2015). Microsatellites allowed distinction between the five entities. Cytological studies were restricted to one representative (F2) acting as a reference dataset (Drosopoulou, unpublished data).

Adult morphology and morphometry, pheromone, cuticular hydrocarbon and distributional data were collected that provide evidence for the specific status of all three formerly recognized taxonomic entities within the FAR complex (i.e. *Ceratitis fasciventris*, *Ceratitis anonae*, *Ceratitis rosa*) (Vaníčková et al. 2014, Břízová et al. 2015, De Meyer et al. 2015, Van Cann et al. 2015). More detailed studies were conducted for the two *Ceratitis rosa* types (R1, R2) (adult morphology and morphometry, pheromone, cuticular hydrocarbon, developmental physiology, behavioural, and ecological data), which provided evidence that the two *Ceratitis rosa* types represent two separate species (Tanga et al. 2015, Van Cann et al. 2015, Vaníčková et al. 2015b), of which one (currently referred to as R2) will be formally described. An altitudinal transect in Tanzania, where R1 and R2 occur in sympatry, confirmed that R1 is more tolerant to higher temperatures and R2 better adapted to colder environments (Mwatawala et al. 2015a). For the two *Ceratitis fasciventris* types (F1, F2) these additional studies could not be conducted as laboratory colonies of one of the two types could not be established, preventing experiments on developmental physiology and mating compatibility. Larval morphology did not provide evidence with regard to the specific status, except for *Ceratitis fasciventris* (F2) to some extent (Steck and Ekesi 2015). Moreover, as a spin-off of this research it was shown that characters previously considered diagnostic for differentiation between species and even between the genera *Ceratitis* and *Bactrocera*, proved to be variable.

The knowledge and gaps identified at the start of the CRP, as well as the progress made by the end of the CRP in addressing the gaps identified for the *Ceratitis* FAR complex are summarized in Table 3.

**Table 3.** Baseline and progress on the *Ceratitis* FAR Complex.

Method	Knowledge at CRP start and gaps identified	Progress in addressing gaps identified	Output References
DNA Analysis	Attempts to develop specific diagnostic markers had been made but were so far ineffective. Need for further exploration for markers, especially microsatellites.	Microsatellites were developed for the *Ceratitis* FAR complex. Population genetic structure for the complex revealed five clearly distinguishable clusters: *Ceratitis fasciventris*: F1, F2; *Ceratitis anonae*; *Ceratitis rosa*: R1, R2. Restriction site associated DNA sequencing (RAD-seq) confirmed the robustness of the five genotypic clusters. There is still the need for a diagnostic marker.	Delatte et al. 2013, De Meyer et al. 2015a this issue, Virgilio et al. 2013
Cytology	No data available. Cytology has the potential to provide a diagnostic tool.	Mitotic karyotype and polytene chromosome analysis of *Ceratitis fasciventris* from Kenya showed rearrangements in two polytene arms and differences in the size of mitotic sex chromosomes.	Drosopoulou (unpubl.)
Morphology	Males of three taxa can be distinguished to some extent, however, separation of females is not possible, and larval morphology so far not studied.	After recognition of five clusters (based on molecular work), adult characters were re-examined. Consistent morphological differences were found to distinguish male *Ceratitis rosa* (R1 and R2) and *Ceratitis fasciventris* (F1 and F2). SEM studies for all immature stages were conducted, including several populations of *Ceratitis rosa* (R1 and R2) from different geographical regions. No diagnostic characters could be found to differentiate the different entities (except for *Ceratitis fasciventris* F2).	De Meyer et al. 2015a this issue, Steck and Ekesi 2015 this issue
Morphometrics	No data available, but might have potential for separation of females, in view that adult females cannot be differentiated on morphological characters.	Morphometric studies on all five genotypes allowed resolving to a large extent morphospecies and genotypic clusters. Wing landmarking might represent a possible tool for the diagnosis for species within the FAR complex.	De Meyer et al. 2015a this issue, Van Cann 2013, Van Cann et al. 2015 this issue
Male Lure Response	FAR complex males apparently attracted to trimedlure, however, response of representatives of the *Ceratitis* FAR complex to male lures to be investigated.	EGOlure shown to be a stronger attractant for *Ceratitis rosa* (R1 and R2) than trimedlure. In addition, EGOlure shown to be able to attract *Ceratitis cosyra* in a significantly stronger way than terpinyl acetate.	Mwatawala et al. 2012, 2015a this issue
Developmental Physiology	Need to determine whether there are developmental / physiological differences between the entities recognized.	Marked difference in development and survival in relation to different temperature ranges in the two *Ceratitis rosa* types both in Kenya and South Africa. *Ceratitis rosa* R1 being more tolerant to higher temperature and *Ceratitis rosa* R2 better adapted to colder environments.	Tanga et al. 2015 this issue
Sexual Behaviour	Only some behavioural work on *Ceratitis rosa*, limited for the other entities. Need to determine any behavioural differences between the entities recognized.	Pre-zygotic mating incompatibility studies under semi-natural conditions using populations of *Ceratitis rosa* R1, R2 and *Ceratitis fasciventris* F2 showed clear evidence of reproductive isolation between the two *Ceratitis rosa* types R1 and R2, similar to the reproductive isolation observed between each of them and *Ceratitis fasciventris* F2.	Ekesi et al. (unpubl.)
Pheromone Components	Pheromones of taxonomic entities recognized so far not studied.	Composition of investigated pheromones is different from that of *Ceratitis capitata*, and confirmed differences among the five FAR taxa.	Břízová et al. 2015 this issue
Cuticular hydrocarbons	Cuticular hydrocarbons not studied, but can contribute to resolving the specific status of the taxa within the complex.	Differences detected between the five taxa recognized in the FAR complex. Sexual differences were also found in each species.	Vaničkova et al. 2014, 2015b this issue
Distribution	Need to re-assess whether there are distributional differences between the entities recognized.	*Ceratitis rosa* R1 largely absent from southern part of the African continent and from higher altitudes. *Ceratitis fasciventris* F1-F2 largely separated (western-central versus eastern Africa), although isolated populations of ‘western’ type also found in Angola, Malawi, Tanzania, Zambia.	De Meyer et al. 2015a this issue, Mwatawala et al. 2015a this issue
Host Ranges	Need for additional studies to determine whether there are host range differences between the entities recognized.	*Ceratitis rosa* R1 and R2 do not show major differences in host range except that the hosts from temperate climates (*Pyrus*, *Rubus*, *Coffea*) are predominantly infested by *Ceratitis rosa* R2. Information for the *Ceratitis fasciventris* entities is inconclusive because lack of F1 data.	De Meyer et al. (unpubl.)

### 4.1 *Zeugodacus cucurbitae* Situation Analysis

The melon fly, *Zeugodacus cucurbitae* (initially referred to as Bactrocera (Zeugodacus) cucurbitae), is a major pest of cucurbit crops that has spread from its area of origin (South East Asia) across Africa, Hawaii, the Indian Ocean, Papua New Guinea and the Solomon Islands (Severin et al. 1914, Dhillon et al. 2005). In particular, it causes severe losses in food crops and restrictions to trade for some cucurbit crops.

Some populations were identified in Africa, islands in the Indian Ocean, Hawaii and South East Asia with different host use, which could indicate the existance of very closely related species. Although the SIT has been effectively applied against the melon fly in certain regions (Koyama et al. 2004), this issue needed to be resolved to enable the application of species-specific treatments such as the SIT against all populations in all regions.

### 4.2. Outputs for *Zeugodacus cucurbitae*

In spite of earlier observations and indication of different host-use by *Bactrocera cucurbitae* in different geographic regions, genetic studies using mitochondrial and nuclear markers indicated very low intraspecific variability worldwide. Population genetic studies using microsatellites were able to distinguish five major groups worldwide: African mainland and Seychelles, Réunion and Mauritius, Central Asia, SE Asia, and Hawaii (Virgilio et al. 2010). However no phylogeographic patterns could be discerned using cytogenetics analyses (Zacharopoulou et al. 2011b, 2013) or mitochondrial and nuclear gene fragments (total of 2764 bp) (Virgilio, unpublished data). The invasion history for the species on the African mainland was also reconstructed (Delatte et al. unpublished data).

Cross-mating experiments were conducted at the start of the CRP between populations of Mauritius, Seychelles and a genetic sexing strain of Hawaii and these indicated no mating incompatibility or sexual isolation (Sookar et al. 2013). Given this fact and the genetic assessments, it was decided there was no need for additional cross-mating studies.

Further studies, including host preference and microsatellite markers, did not show any relation between genetic structure and host plants (Virgilio et al. 2010, Sookar et al. 2013). It was concluded that there is no evidence of the existence of host races or cryptic species within *Bactrocera cucurbitae*. However, as a spin-off of the conducted research, recent studies on the higher phylogeny of dacines have shown that the higher taxonomic classification under which *Bactrocera cucurbitae* is placed, is a paraphyletic grouping, requiring a taxonomic change in generic placement (Krosch et al. 2012, Virgilio et al. 2015). A nomenclatorial act has raised the subgenus *Zeugodacus* (as well as other subgenera belonging to the *Zeugodacus* group, sensu Drew 1989) to genus level. As a result, *Bactrocera cucurbitae* was put in a new generic combination: *Zeugodacus cucurbitae*, and should be referred to by this name from now onwards (Virgilio et al. 2015).

The knowledge and gaps identified at the start of the CRP, as well as the progress made by the end of the CRP in addressing the gaps identified for *Zeugodacus cucurbitae* are discussed in De Meyer et al. (2015b) and summarized in Table 4.

**Table 4.** Baseline and progress on *Zeugodacus cucurbitae*.

Method	Knowledge at CRP start and gaps identified	Progress in addressing gaps identified	References
DNA Analysis	General information on phylogeography / population genetics available on a worldwide basis. Also information available on population genetics with regard to the African mainland and La Réunion. More data on potential host races needed, both within Cucurbitaceae (cultivated versus wild) and cucurbits versus non-cucurbits.	Detailed DNA studies on population genetics in La Réunion, and to a lesser extent in Tanzania, have shown that there is no relation between genetic structure and host plants. The same observation was made from extensive sampling of potential hosts (including non-cucurbits) in Thailand. Mitochondrial variation in *Zeugodacus cucurbitae* populations from Hawaii were studied and little variation detected. Population sampling for African continent increased to retrace the invasion routes and invasion history within and towards/from Africa. The results show that there is a single recent (20^th^ century) invasion event and that the western African populations are the result of a subsequent invasion from eastern Africa.	De Meyer et al. 2015b this issue, Jacquard et al. 2013, Clarke et al. (unpubl.), Delatte et al. 2015 (in press)
Cytology	Work has been carried out on two *Zeugodacus cucurbitae* populations regarding karyotyping and polytene maps.	No cytogenetic differences found between two populations of *Zeugodacus cucurbitae* (genetic sexing strain Hawaii and Bangladesh wild type). Karyotyping reveals that *Zeugodacus cucurbitae* is significantly different from other *Bactrocera* (i.e. position of subgenus *Zeugodacus* in relation to *Bactrocera* and *Dacus*).	Zacharopoulou et al. 2011b, 2013
Morphology	*Zeugodacus cucurbitae* can be morphologically differentiated from other species within the subgenus. Some populations appear to indicate very closely related species.	No other valid taxa have been identified that could cause possible confusion with *Zeugodacus cucurbitae*. Also no differences identified among populations. Therefore no need for further studies. Subgenus *Zeugodacus* erected to genus level.	De Meyer et al. 2015b this issue, Virgilio et al. 2015
Sexual Behaviour	Good knowledge of mating behaviour through studies in Japan, Hawaii and recently in Austria.	Compatibility studies under semi-natural conditions investigating cross-mating among three populations from Hawaii, Mauritius and Seychelles. No sexual isolation was discovered.	Sookar et al. 2013
Host Range	Well documented as a whole, although possibility of host races and different host range reported in different parts of distribution range	Reared from 17 plant species comprising 10 families covering Cucurbitaceae, Solanaceae, Anacardiaceae, Rutaceae and Myrtaceae. Host ranges were studied in relation to genetic structure. Populations of *Zeugodacus cucurbitae* vary in their preference to host plants. A tomato population exclusively preferred tomato compared to the other host plants.	Mwatawala et al. 2010, 2015b
Cuticular hydrocarbons	No studies yet specifically with regard to *Zeugodacus cucurbitae* cuticular hydrocarbons have been conducted	The composition of the cuticle of virgin males and females – ages 5, 15, 20, 30 after emergence – was analysed by GCxGCMS. The preliminary data demonstrate sex- and age-specific differences.	Vaníčková (unpubl.)

## Conclusion

Following an integrative taxonomic approach, the biology, cytogenetics, ecology, morphology, genetics, and physiology of major pest tephritid cryptic species complexes is now much better understood. This increased knowledge has resulted in formal decisions on the species status of some taxa within these complexes, thus facilitating international horticultural trade and simplifing SIT application against pest species of these complexes. In the case of *Anastrepha fraterculus* it was shown that it consists of a complex of a number of different species of no monophyletic origin, with distinct geographic and ecological distributions in Latin America. Also for the *Ceratitis* FAR complex evidence has been provided for the existence of five different entities within this complex from the African region, i.e. *Ceratitis anonae*, *Ceratitis rosa* (R1 and a new species referred to as R2), while for *Ceratitis fasciventris* the biological limits between F1 and F2 are not fully resolved. On the other hand the Asian/African pest fruit flies *Bactrocera papayae*, *Bactrocera philippinensis* and *Bactrocera invadens* were shown to represent populations of *Bactrocera dorsalis*, while only *Bactrocera carambolae* remains a valid species for which molecular and pheromone markers are now available to distinguish it from *Bactrocera dorsalis*. Finally studies among populations throughout the geographic range of *Bactrocera cucurbitae* in Africa and the Asia/Pacific region showed no evidence for the existence of host races. However, the higher taxonomic classification under which *Bactrocera cucurbitae* is placed was found to be a paraphyletic grouping, requiring the elevation of the subgenus *Zeugodacus* to genus level. As a result, *Bactrocera cucurbitae* was put in a new generic combination: *Zeugodacus cucurbitae*.
